# Ranking of Molecular Biomarker Interaction with Targeted DNA Nucleobases *via* Full Atomistic Molecular Dynamics

**DOI:** 10.1038/srep18659

**Published:** 2016-01-11

**Authors:** Wenjun Zhang, Ming L. Wang, Steven W. Cranford

**Affiliations:** 1 Laboratory for Nanotechnology In Civil Engineering (NICE), Boston, MA 02115 United States; 2 Interdisciplinary Engineering Program, College of Engineering, Northeastern University, Boston, MA 02115 United States; 3 Department of Civil & Environmental Engineering, Northeastern University, Boston, MA 02115 United States.

## Abstract

DNA-based sensors can detect disease biomarkers, including acetone and ethanol for diabetes and H_2_S for cardiovascular diseases. Before experimenting on thousands of potential DNA segments, we conduct full atomistic steered molecular dynamics (SMD) simulations to screen the interactions between different DNA sequences with targeted molecules to rank the nucleobase sensing performance. We study and rank the strength of interaction between four single DNA nucleotides (Adenine (A), Guanine (G), Cytosine (C), and Thymine (T)) on single-stranded DNA (ssDNA) and double-stranded DNA (dsDNA) with acetone, ethanol, H_2_S and HCl. By sampling forward and reverse interaction paths, we compute the free-energy profiles of eight systems for the four targeted molecules. We find that dsDNA react differently than ssDNA to the targeted molecules, requiring more energy to move the molecule close to DNA as indicated by the potential of mean force (PMF). Comparing the PMF values of different systems, we obtain a relative ranking of DNA base for the detection of each molecule. Via the same procedure, we could generate a library of DNA sequences for the detection of a wide range of chemicals. A DNA sensor array built with selected sequences differentiating many disease biomarkers can be used in disease diagnosis and monitoring.

Through the relative efficiency of exchanging nucleobases to vary its sequence^1^,^2^,^3^,^4^, DNA provides an ideal platform for diverse molecular engineering. Indeed, it is through this sequence variation that all biological cells and tissues arise. From a technological perspective, the interactions between DNA and small molecules have been exploited to build biochemical sensors for disease diagnosis^5^,^6^,^7^,^8^,^9^ and detection of explosives^10^,^11^, and they have demonstrated very high chemical sensitivity, molecular selectivity and good stability. However, the particular sequences used in these studies were either from earlier experience or literature reports. The question arises: given an arbitrary target molecule, can an ideal DNA sequence be designed to maximize interaction? Here, we tackle the first step of this question, probing the interaction of small molecules with target nucleobases – *e.g.*, the basic building blocks of single-stranded and double-stranded DNA – *via* a computational screening approach. This is the first necessary step in sequence optimization, to be used as a protocol for DNA sequence design *in silico* for specific applications. To the best of our knowledge, no other group has reported how to design DNA sequences to achieve the best detection results for particular molecules.

The molecules of interest we select for the current study are common *biomarkers* – measurable indicators of pathological conditions generated by the body. For example, for diabetes the most common biomarker is glucose levels in blood (which effectively defines the metabolic pathology). However, both acetone^12^,^13^,^14^ and ethanol^14^,^15^ are biomarkers for diabetes found in *breath*. Acetone, for example, is reported to be less than a few hundred ppb (by volume) in the breath of healthy individuals^16^ while for diabetic patients, acetone concentration can reach 560 ppm or even > 1000 ppm^17^. In addition, hydrogen sulfide (H_2_S) is a probable indicator of bad breath, and more importantly, a potential biomarker for a variety of cardiovascular diseases^18^ and chronic pancreatitis^19^. Finally, hydrogen chloride (HCl) is such a toxic gas in the air that exposure to 1.8 ppm of it can cause subject’s upper respiratory system symptoms of sore throat and nasal discharge, and 20 ppm is recommended as a level beyond which can cause severe adverse effects^20^. DNA sensors which are capable of detecting these chemicals with high sensitivity and selectivity substantiate a great potential in disease diagnostics and monitoring, as well as in air quality monitoring.

Pairing nucleobases to molecules will enable the possibility to map an array of DNA sequences for reliable detection of several particular biomarkers of one specific disease, and provides a new paradigm of design, development, and application of advanced engineering material systems, combining computational approaches, optimization methods, and DNA informatics. To this end, full atomistic molecular dynamics (MD) can play a major role in this process, acting as a high resolution “virtual microscope” to characterize DNA/biomarker interactions with high fidelity, complementing experimental results^21^,^22^. This is in line with the current Materials Genome Initiative^23^, using computational means as a method to screen materials’ interactions, with a unique biomolecular focus, creating an expanding library or databank of DNA/molecular interactions.

Here, as a first screening protocol to select the promising DNA sequences, we use full atomistic MD simulation, which has been widely applied in biomolecular systems^24^ to probe the molecular interactions between short DNA model systems and small molecules. Similar methods have been implemented to assess the interactions at biomolecular interfaces, ranging from cellulose nanocrystals^25^,^26^ to protein-ligand systems^27^,^28^,^29^. *In silico*, the DNA sequences can be selected, refined, and optimized prior to synthetic efforts, in an efficient manner. Moreover, simulation can efficiently extract interaction parameters (e.g., the energy of interaction, H-bonding, etc.) difficult to measure experimentally^30^,^31^. It is believed that computational methods can be exploited to better interpret the selection, use, development, and discovery of materials, with a goal to achieve rapid and robust acquisition, management, analysis, and dissemination of diverse materials’ data^21^,^31^,^32^. In theory, rather than screening thousands/millions of potential DNA sequence candidates, MD simulation can be applied to select optimized DNA sequences (highest sequence-chemical sensitivity/affinity) to detect one particular chemical. Simulations will not only help bridge the gap between computer simulation and the experiment but also provide unprecedented insight into the behavior of the atomistic interactions between DNA and molecules.

Specifically, we implement MD simulations to screen binding/unbinding affinity of the four introduced biomarkers (acetone, ethanol, hydrogen sulfide, and hydrogen chloride) to the four nucleotides (A, C, G, and T) on both ssDNA and dsDNA. We note the intent is neither to directly quantify the precise energetics of interaction nor to quantify the specific interaction pathways (which could be the focus of future work). Rather, we wish to demonstrate a screening/ranking procedure, to select the “best” nucleobase for biomarker interaction. From this base, an optimized sequence can then be initiated, leading to sequence-specific DNA sensors. In the following sections, we will introduce the MD simulation method in detail, the yield quantitative information about the binding potentials of the DNA-molecule systems, and important insights we have obtained into the biological processes.

## Methods

Molecular dynamics (MD) is a computational method describing equilibrium and dynamics properties of an atomistic system. It generates the configurations of a system by the integration of Newton’s laws of motion with the time dependence of the molecular system, and also provides information at the microscopic level - e.g., atomic positions, velocities and energetics. It helps to understand the properties of assemblies of molecules in terms of their structures and the microscopic interactions between them. MD serves as a complement to conventional experiments, enabling us to learn something new, something that cannot be found out in other ways. For example, it is near impossible to bind a small molecule with a specific nucleobase in a random DNA sequence experimentally, but relatively easy by simulation. We proceed to describe the molecular models, the MD force field, and the interaction assessment procedure *via* steered molecular dynamics (SMD).

### Molecular Model

**Figure 1:**
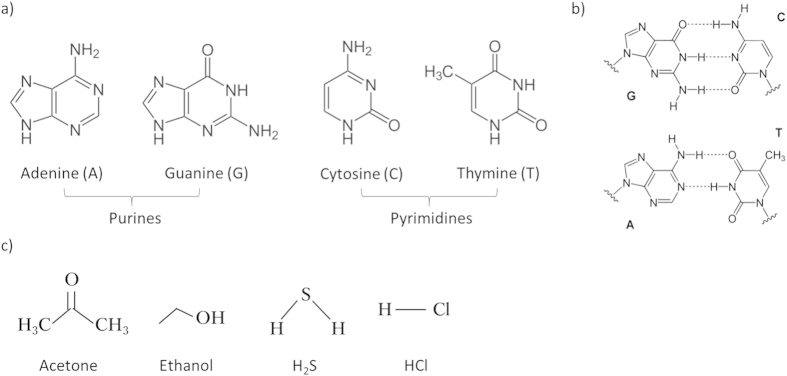
Chemical Schematics. (**a**) structures of DNA bases-A, G, C and T; (**b**) DNA base pairs-G≡C and A = T; and (**c**) structures of chemicals-acetone, ethanol, H_2_S and HCl.

The first step in MD simulation is an accurate construction of the atomistic geometry including a clear definition of the atomistic location, the element type (i.e., the atomic mass and associated chemical properties), and the bond connectivity among all atoms. In our study, it includes single- and double-stranded DNA structural arrangements consisting the definition of each individual nucleotide, targeted biomarker, and surrounding water molecules. DNA (deoxyribonucleic acid) is a long linear polymer built up from many monomer units called nucleotides, consisting of three components: a sugar, a phosphate, and one of the four bases-either adenine (A), guanine (G), cytosine (C), or thymine (T). The backbone of DNA strands is made from alternating phosphate and deoxyribose, so we would use symbols- A, G, C, and T to represent the four types of nucleotides. Two of the bases are derivatives of purines-A and G and two of pyrimidines-C and T (Fig. 1a).

By definition, ssDNA is a single strand lacking base pairs, while dsDNA has two chains winded in a helical structure with the base pairs-G with C and T with A on the sugar-phosphate backbones (Fig. 1b). The double helix is more stable due to the hydrogen bonds between nucleotides and base-stacking interactions among aromatic bases^33^. Both constructed DNA systems consist of 24 nucleotides, constructed in a random manner with the ssDNA sequence: GTCTTACGCTAGCTGGGCATTACG. The dsDNA has one strand with the same sequence and the other of its complementary sequence. The sequence is consistent for all simulations. Structurally, we impose the native helical structure of dsDNA on both models. While ssDNA has been observed to undertake multiple turn and/or hairpin structures^34^,^35^ – and demonstrated to affect ligand binding^36^ - we wish to provide a consistent model for comparison of nucleobase + biomarker interactions. Structural variations of ssDNA strands are left to future works.

The molecules we selected (Fig. 1c) are not only biomarkers for diseases or indicator of air quality but also belong to different functional groups which can greatly help us sum up general rules in the interactions between DNA nucleotides and small molecules. Acetone is the simplest ketone which has two methyl groups at the two ends of a carbonyl group (C = O) forming a nucleophilic property at the oxygen end and an electrophilic property at the sp^2^ hybridized carbon end. It is a hydrogen-bond donor. Ethanol is a 2-carbon alcohol with a nonpolar end (CH_3_) and a very polar end (OH) which enables hydrogen bonding. Hydrogen sulfide is a polar molecule but with very low solubility. Hydrogen chloride is very soluble partly because of its high polarity. These molecules were constructed manually based on known chemical geometry.

**Figure 2:**
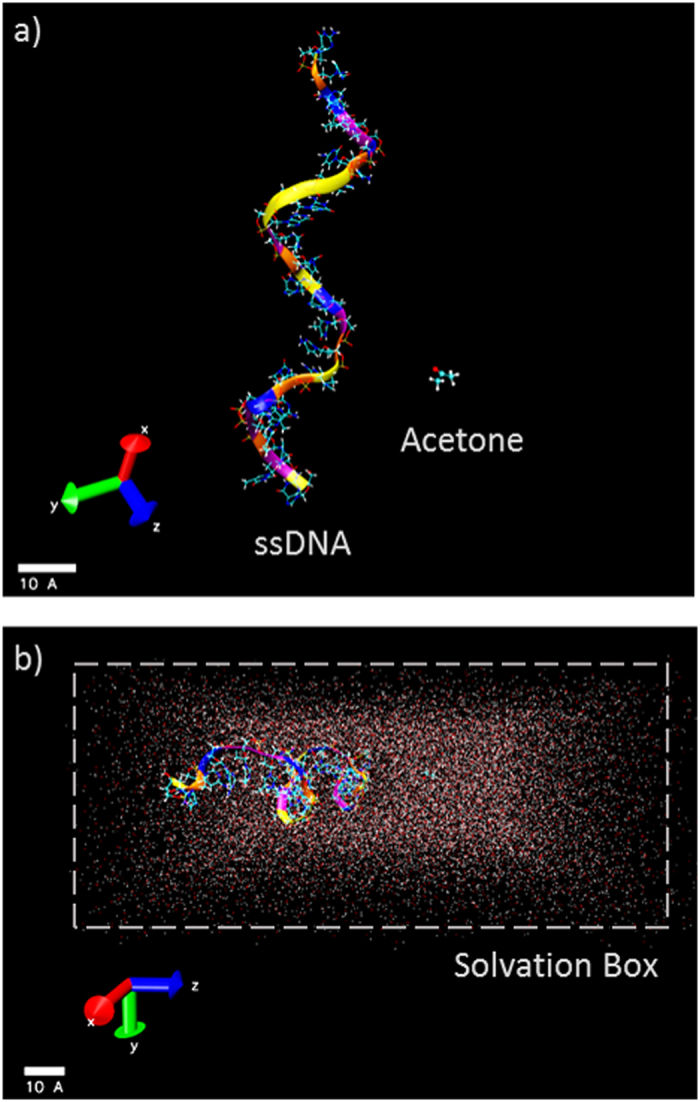
Visualization of the simulated system. (**a**) Full atomistic model of 24 nucleotide-DNA strand-small molecule (Acetone in particular); (**b**) Complete simulation system, with 40 Å × 90 Å × 90 Å periodic water solvation box consisting of 10, 815 water molecules.

Once the molecules were assembled in a single system (Fig. 2a), the ssDNA or dsDNA with biomarker were solvated in a waterbox of approximate dimension 40 Å × 90 Å × 90 Å using the explicit TIP3P water model - a three-site rigid molecule^37^ - as implemented in CHARMM^38^. The large size of the water box is constructed to make sure the whole system is solvated/saturated during the simulation period (Fig. 2b) with adequate screening. The net charge was indeed neutralized by modifying the charges of some solvent molecules to allow long-range solver (e.g., Ewald summation). Note that no explicit counter-ions are included in the current simulation.

It has been known since the first experimental studies on DNA structure that both solvent and counter ions play a major role in stabilizing the double helix and in determining its overall conformation. Indeed, counter ions are critical to maintain the structure of DNA (as well as RNA, and other protein structures). However, here, the ends of the DNA are fixed to prevent any large conformational changes during the simulations. While counter ions have been known to condense around large polyanions such as DNA, the sequence modeled here is too small to reflect the physiological role of counter ions for both stability and neutralization. Typical salt concentrations on the order of 200 mM would require less than 40 molecules be added to a system of over 30,000 atoms. Randomly and homogeneously placed, they would not influence the DNA structure or the nucleobase-biomarker interaction. Moreover, the type of counter ion influences the DNA interactions^39^ and considering the DNA maintained a stable structure, the addition of counter ions was neglected. The effect of such ions, however, can be presumed to provide a screening environment^40^,^41^ for the interactions between nucleobase and biomarker (which are not explicitly charged). Neglecting direct steric interference, the screening effect can be considered near-equal across the biomarkers, and the ranking of interactions thus marginally effected. Of course, this would not be the case for ionized biomarkers.

### Atomistic Force Fields (CHARMM/CVFF)

There are multiple possible force fields (potentials) available to evaluate the inter-atomic interactions which describe the chemical properties and they play an important role in the accuracy of the computational modeling studies *via* proper description of the atomic interactions. In spite of many force fields available in the literature for different types of interactions among atoms, in terms of DNA, we select the well-proven CHARMM force field - a nonreactive potential with a basis on harmonic potentials^42^,^43^,^44^. CHARMM has been parameterized to reflect the structure of DNA^45^,^46^. For the biomarkers, we use parameters from the consistent valence force field (CVFF), which has also been applied in the simulation of polymers, nucleic acids, and organic molecules^47^. The formulation of both CHARMM and CVFF are similar - enabling seamless integration - where the total energy of the system is represented as a sum of covalent (bond, angle, dihedral, and improper) and noncovalent (van der Waals, Coulombic) contributions:


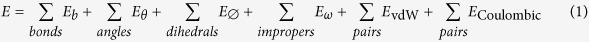


The pair potential parameters of the van der Waals interactions (Lennard-Jones pair potential) between different atom types are mixed according to the geometric mean and arithmetic mean for the energy and distance respectively (e.g., so-called Lorentz-Berthelot scheme):


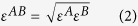



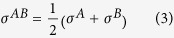


Hydrogen bonds are implicitly included in the Lennard-Jones 12:6 formulation. Both CHARMM and CVFF parameterizations utilize harmonic potentials for covalent interactions such as bond, angle, dihedral, and improper terms with the hypothesis that inter-molecule interactions are significantly weaker than that of covalent bonds. In terms of structural conformation and absolute energies, many MD investigations have been shown to be force-field dependent^48^,^49^. If we implemented a different force field, we would anticipate nominal changes in the quantitative results as the total energies using different potentials are somewhat arbitrary. However, the desired interaction *rankings* would likely remain unchanged. That being said, it is well to keep in mind that no force field is specifically parameterized for current system DNA + biomarker system, and thus subject to interpretation.

For all simulations, Large-scale Atomic/Molecular Massively Parallel Simulator (LAMMPS; http://lammps.sandia.gov/), an open-source molecular dynamics software package is used^50^,^51^. After manually constructing the DNA-chemical system geometry, a minimization of energy for the entire system is done prior to dynamic simulation using a conjugate gradient algorithm to ensure a lowest total potential and stable initial structure. After minimization, unconstrained molecular dynamics simulation over 100ns at 300K using a NVT ensemble is performed to equilibrate the system prior to initiation of SMD. The constraints of the simulation are then be defined and applied to the system to control the simulation process where the computational experiment can be accurately performed.

### Steered Molecular Dynamics (SMD)

To induce interactions between DNA with targeted molecules we implement a non-equilibrium steered molecular dynamics (SMD) approach, which approximately mimics an AFM nanomechanical loading experiment by applying a directional spring force to an objective molecule. SMD is a novel approach to study the dynamics of binding or unbinding events in biomolecular systems^52^, revealing the details of molecular interactions in the course of unbinding^53^,^54^ and providing important insights of the binding mechanisms underlying these processes. The primary advantage of non-equilibrium SMD over conventional equilibrium MD methods is the possibility of inducing relatively large conformational changes in molecules within the nanoscale time scales accessible to computation.

**Figure 3:**
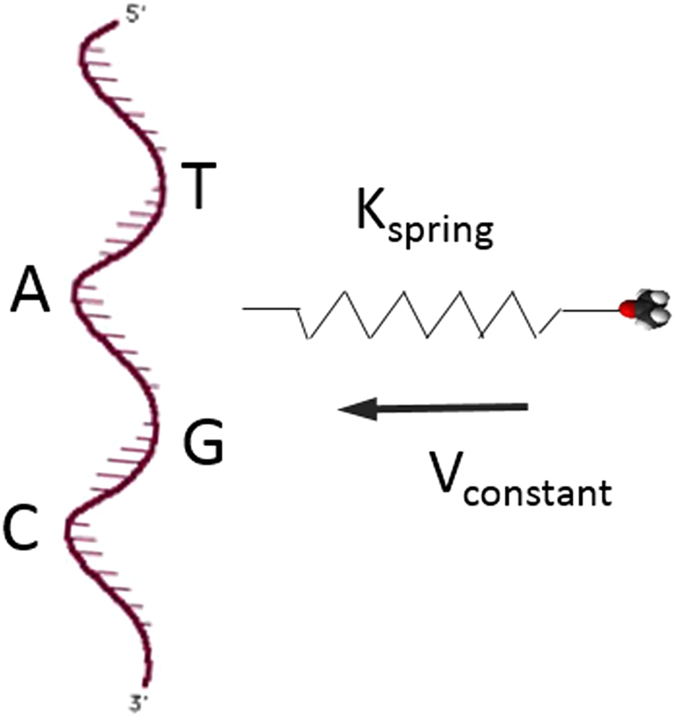
Schematic of SMD simulation. General constant velocity SMD approach where macromolecule is connected with harmonic spring with defined stiffness, k_spring_, and a fixed velocity, v_constant_, towards a target coordinate (x, y, z); in this case, the target is a single nucleobase (A, C, G, T) of a 24-based ssDNA/dsDNA.

Computationally, the SMD method applies a moving spring force (Fig. 3) so that the molecule can behave in a manner not obtained by either force or displacement loading alone, allowing induced conformational changes in a system along a prescribed reaction vector. The driving force applied to the atom group is:


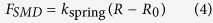


where *k*_spring_ is the spring constant, and *R*_0_ is the distance from the end of spring to an arbitrary tether or target point. A constant velocity, *v*, is assigned which monotonously increments or decrements the distance *R* towards the tether point. The DNA molecule, either single-stranded or double-stranded, is set at one end of the solvation box, and the SMD force is applied at the geographical center atom of the biomarker. As the

DNA molecule is relatively large, its movement during the simulation can be neglected. The small molecules are pulled towards the middle of one particular nucleotide each simulation, providing the direction of spring velocity. Total force and the PMF values during the SMD simulations can then be plotted against the distance between the biomarker and DNA.

**Figure 4:**
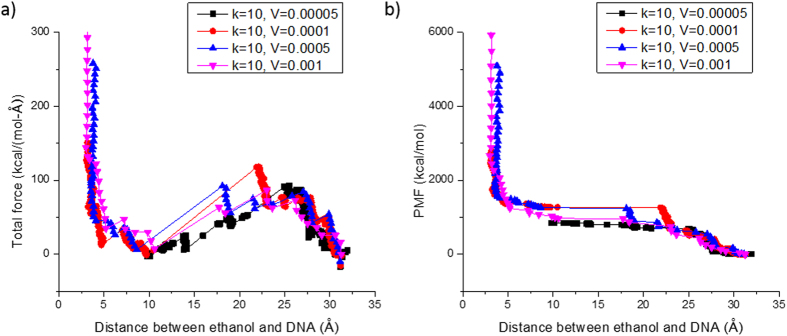
SMD simulation results of G-nucleotide in ssDNA-ethanol system at k_spring_ = 10 kcal/mol/Å^2^ with various pulling speed from 0.00005 to 0.001 Å/fs. (**a**) the total force in the direction of pull; (**b**) the accumulated PMF.

We first decide an appropriate spring constant at which varying the velocity of the spring doesn’t change the applied force and yield PMF values. We can then maintain the constant spring constant and assign a modest pulling velocity throughout the investigation for computational efficiency. It is known that the spring constant can affect the total energy landscape in physical systems^55^,^56^, however, this effect is not studied further here. To select an appropriate spring constant in the SMD simulation, k_spring_, we targeted a G-nucleotide on ssDNA-ethanol system to test different *k*-values at various pulling velocities. An ethanol molecule was pulled close to a G nucleotide on a single DNA strand at different velocities with the k_spring_ value varied as well. For example, at k = 10 kcal/mol/Å^2^, the pulling speed was changed from 0.00005 to 0.001 Å/fs and the total force and PMF values were recorded and compared. After testing different *k*-values ranging from 0.1 to 10, we found at *k* = 10 kcal/mol/Å^2^, the applied force and yield PMF values were about the same when varying the velocity of the spring (see Fig. 4). For the total force, a clear drop was observed from approximately 23 Å to 10 Å, where the interaction between the two molecules started. The more the applied force drops, the stronger attraction force between the two molecules indicating a higher affinity between them. Once the distance between them became very small, less than 5Å, the two molecules started repelling each other leading to an exponential increase of both total force and the energy. The total force (Fig. 4a) and accumulated PMF (Fig. 4b) provided almost the same profiles at different pulling velocities ranging from 0.00005 to 0.001 Å/fs. Thus, we use *k*_spring_ = 10 kcal/mol/Å^2^ (6.95 N/m) and *v*_constant_ = 0.0001 Å/fs (10 m/s) as the setup for SMD simulation.

### Jarzynski Equality and Biomarker Interaction Ranking

Here, we wish to probe the interaction between a biomarker. Despite plentiful modeling methods for such interactions^57^, little is known *a priori* about processes of binding and unbinding, limiting any predictive (or design) power. Presently, the prevailing point of view concerning computer simulations describing binding and determining binding affinities is to strive for the ideal of reversibility, as in umbrella sampling and free energy perturbation^58^,^59^,^60^,^61^,^62^,^63^,^64^, with the hope that artifacts induced by the finite rate of conformational changes can be neglected. Reaching this ideal, however, requires extremely slow manipulation and, therefore, prohibitively expensive simulations. An SMD simulation is a non-equilibrium process, which accepts irreversibility, ceding for the present time accurate evaluation of binding affinities and PMFs, but gaining access to biologically relevant information related to non-covalent bonding. PMF can be equated to the free energy profile along the reaction path and is determined through the Boltzmann-weighted average over all degrees of freedom. It is calculated via the numerical integration of the forces over distance (work) per timestep. With all the other degrees of freedom averaged out, the progress along the reaction vector (e.g., energy across reaction path) is more accurately described.

The concern that thermodynamic potentials cannot, even in principle, be obtained from irreversible processes has been proven unfounded by the *Jarzynski Equality*^65^. The Jarzynski Equality (JE) is a statistical mechanical equation that relates the change in free energy, Δ*F*, between two equilibrium states *via* a non-equilibrium process. Here, this is a bound biomarker (state A) with an unbound biomarker (state B) *via* SMD. In a quasi-static process, the work, *W*, done on a system from 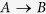 can be said to be:


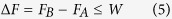


when the system transitions from A to B infinitely slowly. The JE, on the other hand, remains valid regardless of the process speed, where:


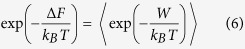


where 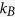 is Boltzmann’s constant and *T* the temperature. The angled brackets, 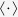, indicates an average over all possible realizations of the external process that takes 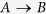. This identity connects the ensemble average of an exponential of the total work *W* performed on the system during a non-equilibrium transition from one state to another to the free energy difference Δ*F*, which is an equilibrium property, between the two states. In general, *W*, depends on the specific initial micro- (or nano-) state of the system, where the ensemble of multiple states implies 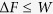. Since its derivation, the JE has been verified to be an accurate (although non-exact^66^) approach in both experiments^67^ and simulations^68^ of biomolecules and small molecules.

To properly capture the free energy describing the conformational space of the binding event and ensure the equality in Eq. (6), the proposed SMD simulations would need to include a very large statistical sample of both multiple initial conditions and multiple directions of the binding vector. This would enable an accurate calculation/prediction of Δ*F*. For *ranking* purposes, however, this degree of accuracy is unnecessary. The JE implies an average of the work over all phase space trajectories from one state to another. Here, we probe one approach/trajectory per biomarker/nucleobase pairing. However, the trajectory is *equivalent* for all systems. Thus, we assert that we apply an equivalent microstate between systems, while Δ*F* cannot be accurately determined, *W* can be sufficiently approximated to rank the attained approximate energies, 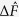, of the interactions. In simple terms, asserting the same initial conditions will result in similar deviations in free energy, such that the ranking of Δ*F* and 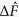 for each biomarker/nucleobase pair does not change.

## Results and Discussion

**Figure 5:**
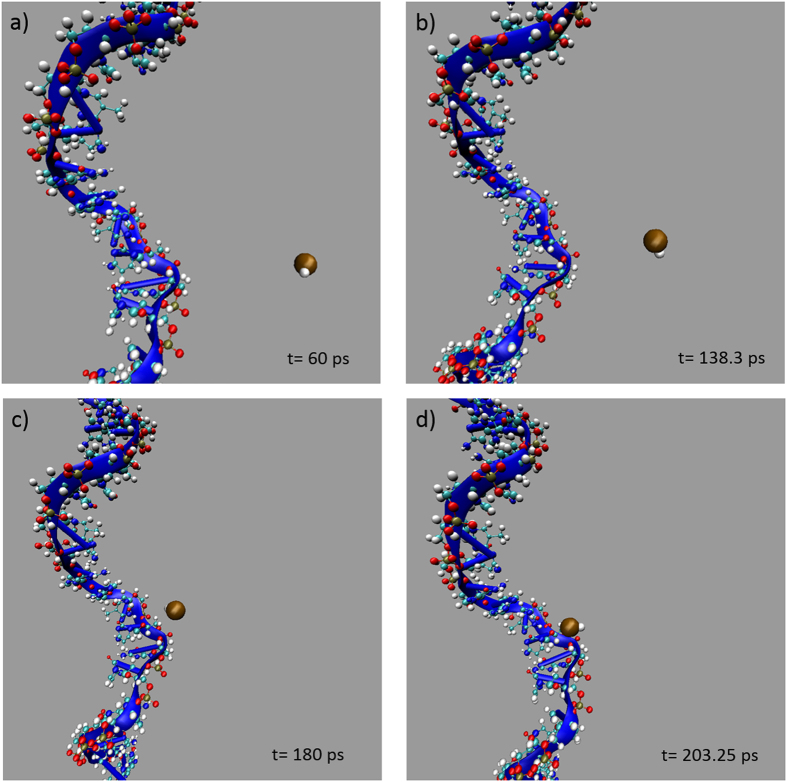
Representative SMD simulation snapshots of G-nucleotide in ssDNA-HCl system at pulling speed of 10 m/s at (**a**) 60 picosecond, (**b**) 138.3 picosecond, (**c**) 180 picosecond, and (**d**) 203.25 picosecond. It is noted that the water molecules with their hydrogen bonds are not shown (but exist all the time) to highlight the interaction between DNA nucleotide and HCl molecule.

**Figure 6:**
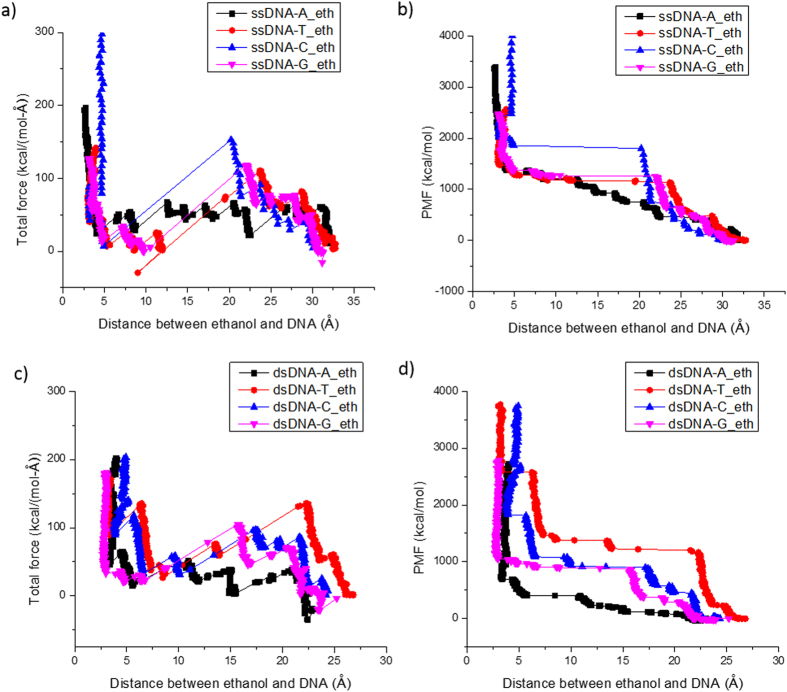
SMD simulation results of A, T, C, and G nucleotides in ssDNA-ethanol system (**a,b**) and in dsDNA-ethanol system (**c,d**) at k_spring_ = 6.95 N/m with pulling speed at 10 m/s. (**a,c**) the total force in the direction of pull; (**b,d**) the accumulated PMF.

We conducted SMD simulations of the interaction between DNA and targeted molecule to investigate the affinity of the molecules on different DNA nucleobases. Specifically, we study the interactions between single nucleotide (A or G or C or T) on both ssDNA and dsDNA with the targeted molecules (acetone, ethanol, H_2_S and HCl), or 32 systems in total. In this paper, we do not show the results of all simulations. They can be found in Supporting Information. The affinity between the DNA nucleotides and chemicals can not only be assessed via energy profiles but also be simply visualized by the geometry. Snapshots of the ssDNA-HCl model (targeting G-nucleotide) are shown in Fig. 5. As a representative example, interactions between four different nucleotides on both ssDNA and dsDNA with ethanol molecule are displayed in Fig. 6a,b and Fig. 6c,d respectively.

### Biomarker Interaction with ssDNA

For different nucleotides applied in ssDNA-ethanol systems, clear force drops were all observed when ethanol molecule was pulled towards T, C and G nucleotides, while the pulling force of ethanol towards A nucleotide almost remained the same until the two molecules started repelling each other (Fig. 6a). It seems ethanol is not attracted to A nucleotide as much as to the other nucleotides according to the force profiles, however, by looking into the accumulated PMF values which reveals the energy profiles, the energy of A, T and G nucleotides and ethanol systems was much lower than that of C nucleotide-ethanol system. Therefore we conclude the affinity of ethanol with different nucleotides on ssDNA follows a trend of T ≥ G > A > C. Ethanol is a polar molecule with a hydroxyl functional group at the end. It can interact with carbonyl and amine groups forming hydrogen bonds. Adenine has three tertiary amines, Guanine has two tertiary amines and one carbonyl group, Thymine has two carbonyl groups, and Cytosine only has one carbonyl group and one tertiary amine groups (e.g., see Fig. 1). It is also known aromatic ring can weaken the electron-donor property of O (oxygen) and N (nitrogen), and primary and secondary amines can form hydrogen bonds with hydroxyl group as well. Therefore it makes great sense that the attraction between ethanol and single-stranded DNA follows a trend of T ≥G > A >C. This simple ranking procedure represents a great feasibility to select the optimal DNA nucleotide/nucleotides towards particular biomarkers without the need of sophisticated computational chemistry (e.g., molecular system energy) or conducting a lot of conventional experiments.

### Biomarker Interaction with dsDNA

For different nucleotides used in dsDNA-ethanol systems, more fluctuations could be seen in the pulling force profiles, and this is due to the complexity of the dsDNA structure which has complementary pairing interactions within the two strands of DNA molecules. Guanine and Cytosine can pair together forming three hydrogen bonds while Adenine and Thymine can form two hydrogen bonds. Since the DNA double helix is stabilized by the hydrogen bonds between the nucleotides and by the base-stacking interactions among aromatic nucleobases, the interactions between nucleotides and ethanol molecules were weakened. This is depicted as the increase of PMF levels compared to that of ssDNA-ethanol system. It is concluded from the energy profiles that the affinity of ethanol with different nucleotides on dsDNA follows a trend of A > G > C > T. Single-stranded DNA is more favored here because of its higher specificity. For instance, one type of nucleotides on one strand of a dsDNA shows the weakest interaction with one particular molecular biomarker while its pairing nucleotide may have the strongest interaction with this biomarker. Thus, the sensitivity and specificity of dsDNA chemical sensors are both reduced compared to ssDNA ones.

**Figure 7:**
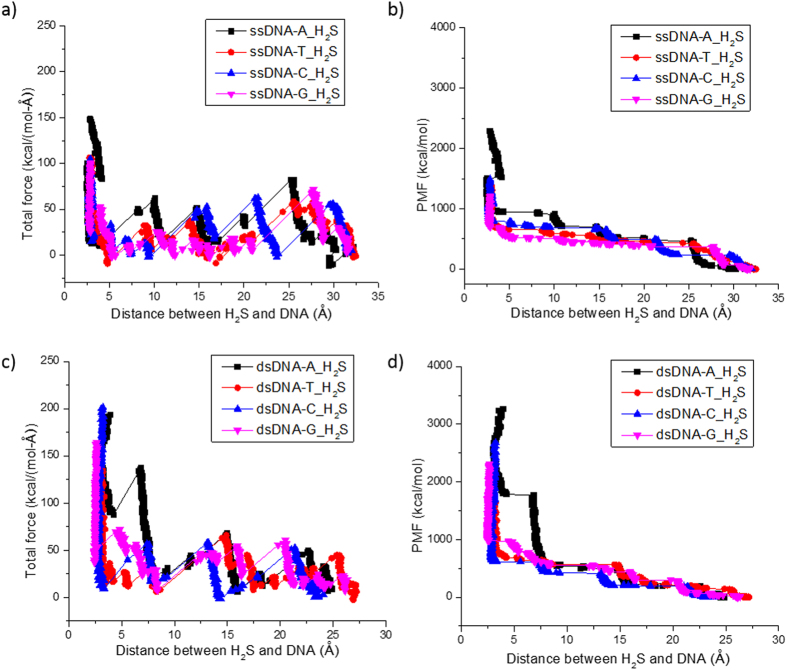
SMD simulation results of A, T, C, and G nucleotides in ssDNA-H_2_S system (**a,b**) and in dsDNA-H_2_S system (**c,d**) at k_spring_ = 6.95 N/m with pulling speed at 10 m/s. (**a,c**) the total force in the direction of pull; (**b,d**) the accumulated PMF.

Similarly, for DNA-H_2_S systems, we obtained SMD simulation results as shown in Fig. 7. H_2_S is a three-atom polar molecule (Fig. 1c), it is much smaller and lighter compared to acetone and ethanol. Thus the interaction between DNA and H_2_S is more active/variable. This behavior may be attributed to the dynamic formation of weak hydrogen bonds with H_2_O and/or DNA molecules^69^,^70^,^71^, which is consistent with the more fluctuations of the pulling force observed in both ssDNA-H_2_S and dsDNA-H_2_S systems, due to the binding competition between the solvent (H_2_O molecules) and the DNA (target nucleobase). Similar behavior was observed in the relatively small DNA-HCl systems.

### Ranking of Biomarker Interaction with DNA

**Figure 8:**
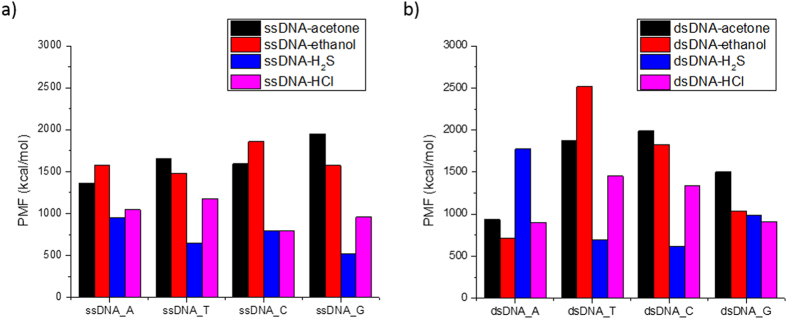
Ranking of molecular biomarker- acetone, ethanol, H_2_S and HCl, interaction with A, T, C, G nucleotides on (**a**) ssDNA; and (**b**) dsDNA indicated by PMF values.

Ranking of the four molecular biomarkers interaction with four different DNA nucleotides on both ssDNA and dsDNA is depicted in Fig. 8, based on PMF results. A similar ranking could theoretically be accomplished for the observed maximum unbinding forces between biomarker and DNA nucleobase. However, we are primarily concerned with the binding affinities (as the intended application is in biomarker detection) and the binding/unbinding forces are not necessarily equivalent, depending on the interaction trajectory^72^. The PMF, however, should remain consistent, due to the averaging of the energetic landscapes^72^.

It behooves us to note the importance of repeatability and scatter in the observed PMF values. As the biomarkers are relatively small molecules, large energetic variation upon repeated runs (which could arise from conformational changes, for example) is unlikely. However, as a trial case (due to the computational time required per simulation), we have tested simulation consistency using the ssDNA-acetone system. From a different starting configuration, the simulation was repeated to attain three total data points for each nucleobase interaction. The variance of PMF values for each mononucleotide-acetone systems is less than 5% of the mean, supporting the reliability of the simulation results, particularly for ranking purposes (see Supporting Information for variations).

Summary of the affinity of four chemical molecules with different DNA nucleotides is provided in Table 1. The criteria applied to generate the rankings include first - the PMF value before the two molecules start repelling each other; second - the closest distance the molecular biomarker can approach DNA nucleotide; and third - the total force drop when two molecules are pulled together. Here we focus more on the interaction between nucleotides on ssDNA with molecular biomarkers due to the stronger interaction and higher specificity as compared to those observed with dsDNA. Adenine nucleotide shows the highest affinity with acetone molecules, and this can be attributed to the carbonyl group (C=O) in the acetone molecule which reacts with the amine group (−NH_2_) in adenine. In spite of the existence of similar amine groups in guanine, cytosine, and thymine nucleobases, the effect of electron-donating is weakened by the carboxyl groups in cytosine and thymine nucleobases, especially by the aromatic ring presented in the guanine nucleobase. Thus, the affinity between acetone and different nucleotides follows the trend A > C ≥ T > G when considering single-stranded DNA.

The interaction between small molecules and double-stranded DNA is much more complicated due to the hydrogen bonds within the dsDNA and its complementary pairing properties. The ethanol molecule has a very polar end- hydroxyl group, for example, which interacts with both carbonyl groups and amine groups forming hydrogen bonds. With the weakening effect from the aromatic ring and the enhancing effect from primary and secondary amine groups, it makes great sense that the affinity between ethanol and single-stranded DNA follows a trend of T ≥ G > A > C. H_2_S is a polar molecule, and S (sulfur) is an electron-rich element and a homologue of O (oxygen). Based on the principle of the dissolution in the similar material structure, adenine nucleotide should have the least affinity with ethanol molecule, and it is also the result from our simulation which confirms the practicality of our computational approach. HCl is very soluble due to its high polarity. It is hard to predict the interaction between HCl and nucleotides, but MD simulation has provided with a clear trend of the affinity between these two molecules.

### Experimental Comparison

Due to a lack of isolated DNA with biomarker systems, we resort to a comparison between the simulated results and a known DNA + carbon nanotube sensor. In our earlier experiments on sensing trace amount of chemicals in vapor^73^, we designed and fabricated a wireless sensor array based on ssDNA-decorated single-walled carbon nanotube (SWNT) on micro-devices. Microelectrodes with 3 μm gap were fabricated by photolithography followed by sputtering Cr/Au (20 nm/150 nm) layer onto a silicon oxide substrate. Then ultrathin films of SWNTs were assembled between pairs of microelectrodes by a low temperature and also low cost DEP assembly process. ssDNA of different sequences were non-covalently bonded to the SWNT surfaces^73^. Changes in resistance indicated the interaction between DNA and gas molecules. The sequences used included:

DNA 24A: AAAAAAAAAAAAAAAAAAAAAAAA

DNA 24Aa: amine-AAAAAAAAAAAAAAAAAAAAAAAA-amine

DNA 24GT: GTGTGTGTGTGTGTGTGTGTGTGT

DNA 24Ma: amine-GTCTTACGCTAGCTGGGCATTACG-amine

By introducing the carbon nanotube, the presence of the hydrophobic nanotube sidewall directly adjacent to the nucleotides (which are adsorbed on the sidewall via their hydrophobic bases) may significantly change the binding environment for a given biomarker – however, we can qualitatively compare the results. Taking acetone sensing as an example, the response ranking of the ssDNA-SWNT sensors towards acetone follows 24Ma > 24A > 24GT > 24Aa, which slightly differs from the simulated ssDNA-acetone interaction result (A > C ≥ T > G, in Table 1). There are two factors which determine the chemical sensing performance using the sensor array. There are binding between SWNT and ssDNA and interaction between ssDNA and chemicals. Khamis *et al.* has reported that the affinity of homo-ssDNA wrapping around SWNT follows a trend of G > A > T > C^74^, and this means ssDNA sequences with more G nucleotides would bind more onto SWNT sensor. Stronger binding between ssDNA and SWNT can create a more hydrophilic environment around the hydrophobic SWNT core and facilitate the adsorption of acetone molecules. On the other hand, the interaction between ssDNA and acetone follows A > C ≥ T > G which means A nucleotide reacts stronger with acetone. DNA 24Ma functionalized SWNT sensor has a sequence of mixed A, G, C, and T nucleotides, and it has demonstrated a stronger response compared to DNA 24A-SWNT sensor. It implies that adding G nucleotides into DNA sequence can improve the acetone sensing performance by increased binding between DNA and SWNT. DNA 24GT decorated SWNT sensor displayed the second least affinity towards acetone, and it is consistent with the simulated ssDNA-acetone interaction ranking. The fact that DNA 24 Aa decorated SWNT sensor revealed the least response to acetone is likely due to the intermolecular interactions between DNA molecules at the amine group ends. Though we don’t have experimental results on all four homo-ssDNA decorated SWNT sensors for acetone sensing, the limited ranking of responses of ssDNA-SWNT sensors on acetone is very comparable to the SMD simulation results combined with the affinity ranking of SWNT and ssDNA. Thus, we would high confidently recommend a DNA sequence consisting most of G and A nucleotides -decorated SWNT sensor for highly sensitive acetone detection based on DNA G nucleotide’s best binding ability to SWNT and DNA A nucleotide’s best interaction with acetone molecule and good affinity to SWNT as well. The combination of DNA and nanotubes *in silico* is to be the focus of future work.

### Unbinding Simulations

A condition of the JE is that is holds for the reversible process – e.g., the work necessary for transition from 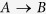 should be equivalent to the magnitude of the work necessary for 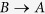, or 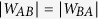. That being said, as before, the equivalence presumes a full statistical sampling of all possible microstates for each transition. Moreover, the initial microstates differ for each transition. Regardless, between biomolecular systems, we again assert that deviations from exact energetic values would be consistent, as each biomarker is subjected to the same microstate, as such the ranking of unbinding simulations should reflect the physical molecular affinities.

**Figure 9:**
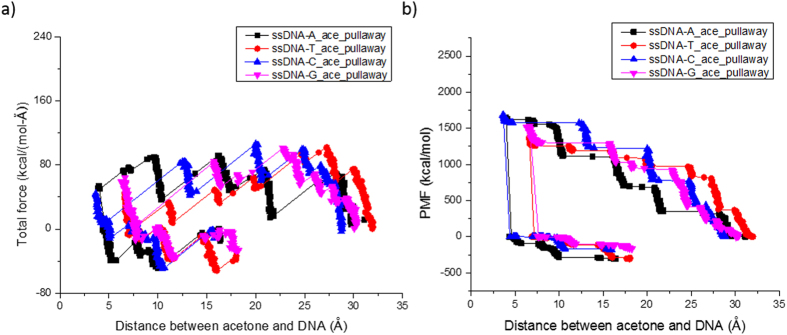
SMD simulation results of A, T, C, and G nucleotides on ssDNA-acetone system at k_spring_ = 6.95 N/m, v_constant_ = 10 m/s with acetone molecule being pulled towards DNA and then pulled away from DNA. (**a**) the total force in the direction of pull; (**b**) the accumulated PMF.

As such, we also simulated the pulling-forward (binding) and following pulling-away (unbinding) processes to confirm the affinity of the different biomarkers with different DNA nucleotides. Taking ssDNA-acetone system for example (Fig. 9), the absolute value of PMF increased when acetone was pushed away indicating there was an attraction force between acetone and DNA (Fig. 9b). A higher absolute value of PMF when pulling acetone molecule away from DNA means a higher affinity between these two molecules. As shown in the lower part of Fig. 9b, the pulled away |PMF| follows the trend A > C ≥ T > G which supports the affinity strength rank obtained through the pulling forward approach (Table 1).

### Effect of Nucleobase Neighbor

**Figure 10:**
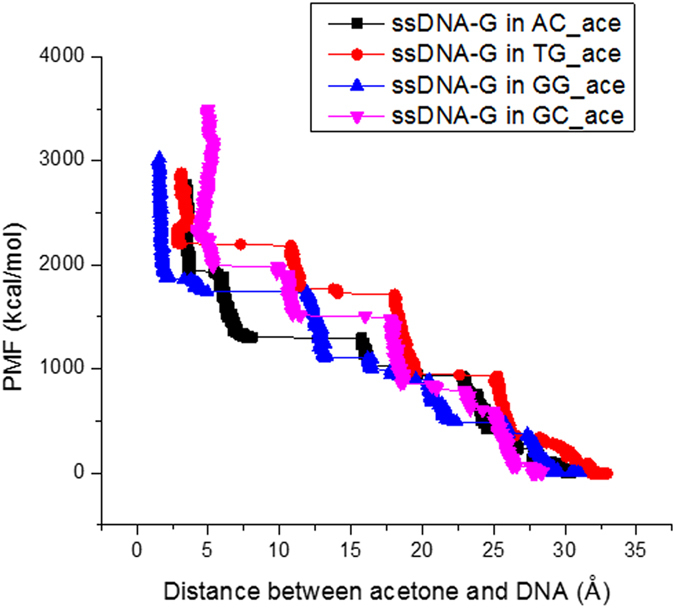
SMD simulation results-the accumulated PMF of G nucleotides with different neighboring nucleotides on ssDNA-acetone systems at k_spring_ = 6.95 N/m, v_constant_ = 10 m/s.

Finally, while we target single nucleobases, the ultimate goal is to optimize the entire DNA sequence for biomarker interaction. Control of neighboring bases would clearly be necessary for larger biomarkers, with concurrent interactions between bases and functional groups. Here, the molecular size of the biomarkers are actually smaller than the target base, and thus one-to-one pairing is presume sufficient for preliminary ranking. That being said, to investigate preliminary effects of sequence variation, we probe the interaction between nucleobases with variant neighbors. As indicated in Fig. 10, for the same nucleotide-G, the PMF values vary on the neighboring nucleotide types. The ssDNA-acetone system with G nucleotide positioned between G-G nucleotides provides the lowest energy at the closest distance while the energy increases when G is positioned between A-C, G-C, or T-G nucleotides. This indicates the interaction between neighboring nucleotides and the studied nucleotide also affects the affinity of the studied nucleotide-small molecule system. Again, the effect of neighbors will be dependent on the size of the selected biomarker, as well as the screening environment (e.g., presence of ions) which is not varied here, and suggests additional future investigation. For example, stretches of three bases (e.g., AAA, GAG, GAT, etc) may be necessary for optimal interaction. This would require a combinatorial approach (requiring extensive simulation sets per biomarker). Further such study of the effect of DNA sequence and length is planned. Validation of the simulation results through conventional experiments is also in progress.

## Conclusion

In order to select the optimal DNA sequences in building DNA biochemical sensors for breath analysis or air quality monitoring, we conducted SMD simulations of the interactions between different DNA sequences and our targeted molecules. Acetone and ethanol are breath biomarkers for diabetes, while elevated H_2_S level can indicate cardiovascular diseases or chronic pancreatitis. HCl is highly toxic and can cause immediate danger to life with only 50 ppm concentration. We studied the interaction between four single DNA nucleotides (A, G, C, and T) on both ssDNA and dsDNA with acetone, ethanol, H_2_S and HCl. In SMD simulation, the center-of-mass of small molecules was pulled at a certain velocity towards one particular DNA nucleotide. The mechanical work of pulling it forwards (forward pulling path) and backwards (reverse pulling path) at a number of points was measured during this process. By sampling these forward and reverse paths, we were able to know the equilibrium distance and to accurately compute the free-energy profiles of the eight aforesaid systems for each targeted molecule. Four DNA nucleotides on dsDNA were found to react differently to the targeted molecules than on ssDNA, requiring significant higher energy to move the molecule close to DNA than the later. Comparing the PMF values of the different systems, we obtained the optimal DNA bases/sequences for the detection of each molecule: Adenine for acetone, Thymine for ethanol, Guanine for H_2_S, and Cytosine for HCl. Taking the affinity ranking of SWNT and ssDNA into account, the simulation results are in good agreement with our earlier sensing results using ssDNA-SWNT sensors. A library of DNA sequences for the detection of a wide range of chemicals can be easily generated *via* this method. A DNA sensor array built with selected sequences differentiating many disease biomarkers or indicating harmful gases in the air can be used in disease diagnosis and monitoring and air quality monitoring as well.

Future work will focus on 1) applying full atomistic molecular dynamics characterization approaches to a subset of DNA sequences (combinatorial) and more selected biomarkers, 2) developing an automated optimization process rather than manually assess all possible sequence variations in a brute-force approach, and 3) conducting experiments for validation. Moreover, a set of simulations implementing more defined sequences is necessary to delineate clear binding mechanisms, and differentiate between interaction contributions (e.g., H-bonding versus electrostatic, for example). Clearly, such an approach is beyond the scope of a single study. Ultimately, we intend to exploit DNA as a tunable material for sensing applications by using computational screening. MD can successfully and systematically provide atomistic details of the binding mechanisms, revealing transient interactions that can be exploited in-depth. Later on, this systematic design methodology can be applied to tailor the behavior of a material system driven by molecular interaction metrics.

## Additional Information

**How to cite this article**: Zhang, W. *et al.* Ranking of Molecular Biomarker Interaction with Targeted DNA Nucleobases *via* Full Atomistic Molecular Dynamics. *Sci. Rep.* **6**, 18659; doi: 10.1038/srep18659 (2016).

## Supplementary Material

Supplementary Information

## Figures and Tables

**Table 1:** Affinity strength rankings of DNA nucleotide-chemical systems.

	Ranked Affinity	Ranked Affinity
Biomarker	with ssDNA	with dsDNA
Acetone	A>C≥T>G	A>G>T>C
Ethanol	T≥G>A>C	A>G>C>T
H_2_S	G>T>C>A	C>T>G>A
HCl	C>G>A>T	G>A>C>T
