# Supplementary material for: Ranking of Molecular Biomarker Interaction with Targeted DNA Nucleobases via Full Atomistic Molecular Dynamics
Source: Sci Rep. 2016 Jan 11;6:18659. doi: 10.1038/srep18659 (PMC4707552; doi:10.1038/srep18659)
Supplement: Supplementary Information [file srep18659-s1.pdf]

Ranking of Molecular Biomarker Interaction with Targeted DNA Nucleobases *via* Full Atomistic Molecular Dynamics

Wenjun Zhang[a,b], Ming. L Wang[a], Steven W. Cranford[a,b*]

[a]*Laboratory for Nanotechnology In Civil Engineering (NICE)*
[b]*Department of Civil & Environmental Engineering, Northeastern University*
* Corresponding author: s.cranford@neu.edu

SUPPORTING INFORMATION

S1. ADDITIONAL PLOTS/SIMULATION RESULTS

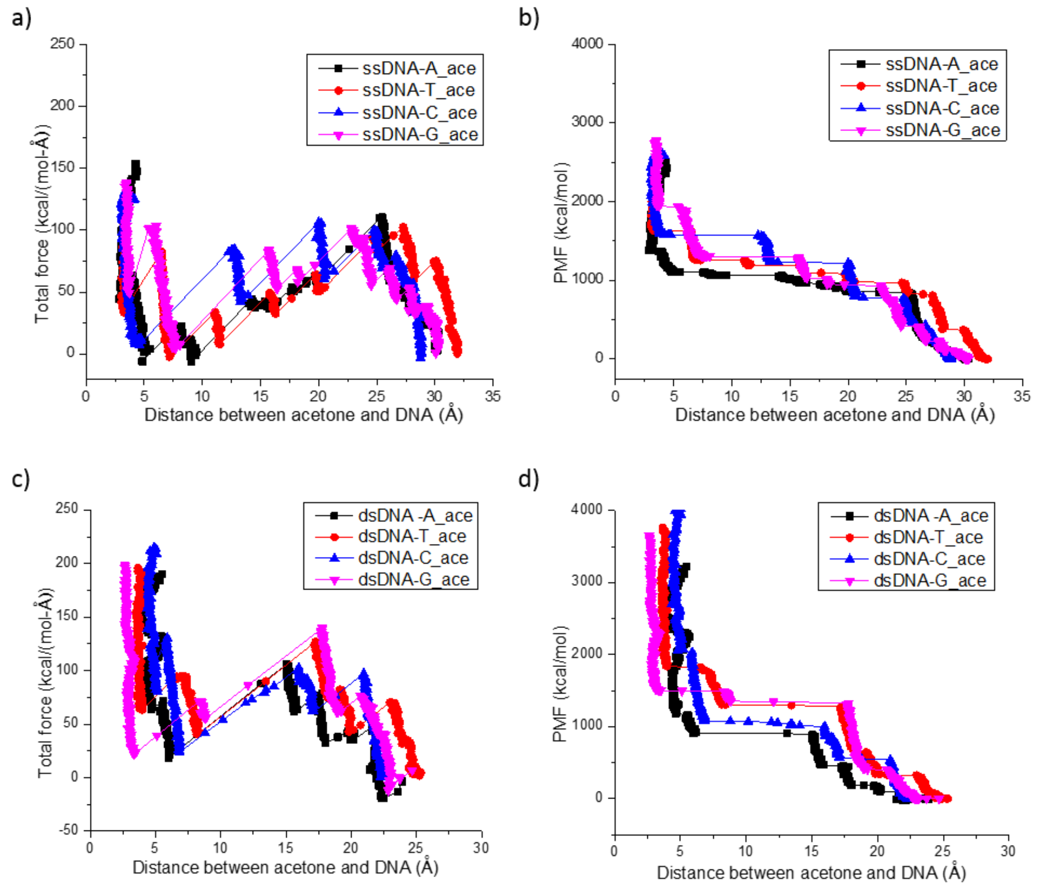

Figure S1: SMD simulation results of A, T, C, and G nucleotides in ssDNA-acetone system (a and b) and in dsDNA- acetone system (c and d) at k_{spring} = 6.95 N/m with pulling speed at 10 m/s: a) and c) the total force in the direction of pull; b) and d) the accumulated PMF.

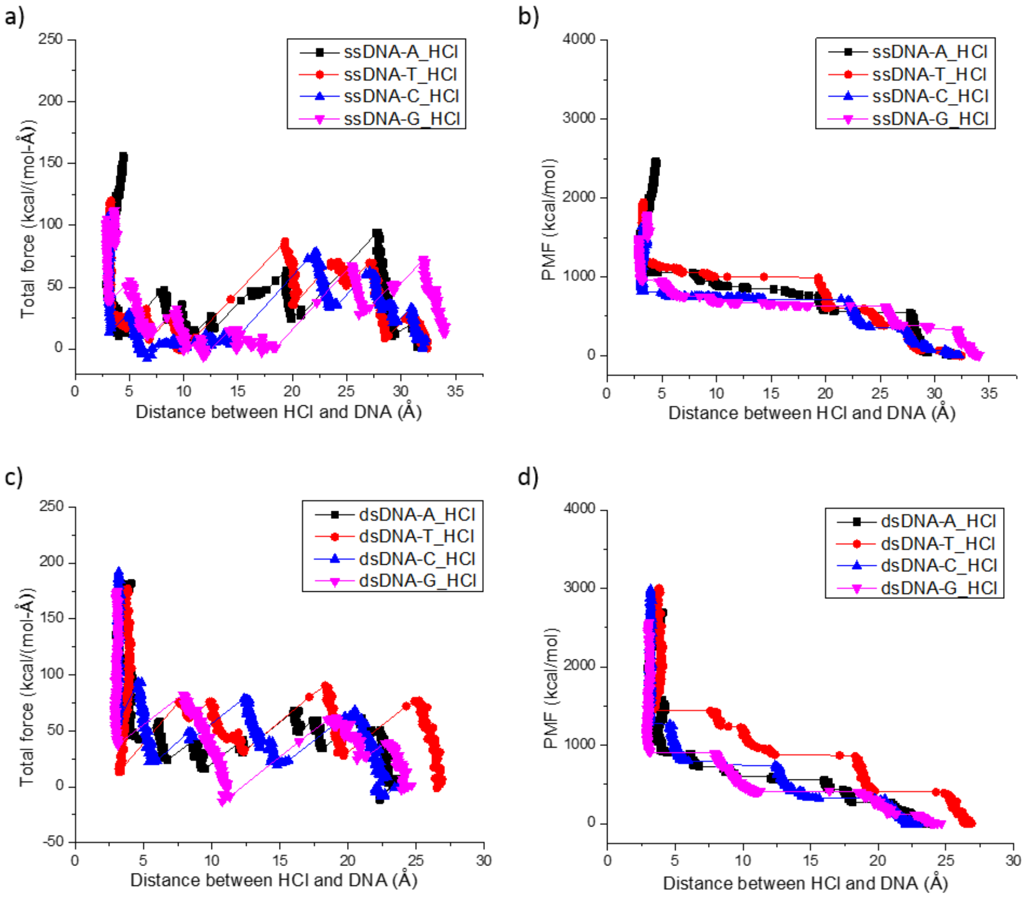

Figure S2: SMD simulation results of A, T, C, and G nucleotides in ssDNA-hydrogen chloride system (a and b) and in dsDNA- hydrogen chloride system (c and d) at k_{spring} = 6.95 N/m with pulling speed at 10 m/s: a) and c) the total force in the direction of pull; b) and d) the accumulated PMF.

S2. Repeatability Trial Case

As the biomarkers are relatively small molecules, we do not anticipate much energetic variation upon repeated runs (which could arise from conformational changes, for example).

As a trial case (due to the computational time required per simulation), we have tested the simulation repeatability using the ssDNA-acetone system.

From a different random starting configuration, the simulation was repeated two additional times (an additional 8 runs resulting in three total data points for each nucleobase).

The small variances of PMF values for each mononucleotide-acetone systems (<5%) confirmed the reliability of the simulation results (see **Figure S3** below).

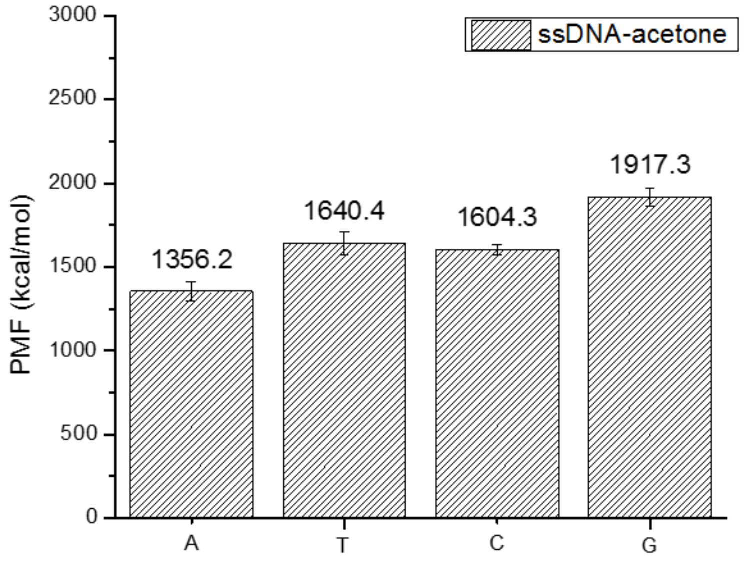

Figure S3. The repeatability of SMD simulations illustrated by PMF values when pulling acetone close to mononucleotide - A or T or C or G on ssDNA. Error bars = ± standard deviation and n = 3.
